# Underground Ferromagnetic Pipeline Detection Using a Rotable Magnetic Sensor Array

**DOI:** 10.3390/s25237153

**Published:** 2025-11-23

**Authors:** Xingen Liu, Zifan Yuan, Mingyao Xia

**Affiliations:** School of Electronics, Peking University, Beijing 100871, China; xingenliu409@stu.pku.edu.cn (X.L.); zfyuan@stu.pku.edu.cn (Z.Y.)

**Keywords:** underground pipeline, magnetic sensor array, rotating scanning, location, orientation estimates

## Abstract

To eliminate the risk of damage to buried pipelines during excavation, a survey in advance or on the spot is necessary. Here we propose a wireless rotable magnetic sensor array to detect underground ferromagnetic pipelines. It consists of several sensing nodes placed on a rail, which can rotate automatically or manually. We adopted rotating rather than translating the array since translation is difficult on uneven or muddy ground. Moreover, we could judge the existence and orientation of a pipeline by simply checking the periodic variation of measured data without resorting to complex inversion algorithms. Field experiments showed that the equipment could provide a decimeter-level locating accuracy for both the horizontal offset and buried depth, and a strike angle error of a few degrees, which meet general engineering application requirements.

## 1. Introduction

Damage to underground utilities, such as gas and sewer pipelines, can result in costly financial losses, service interruptions, and even serious injury or loss of life [1]. As urbanization accelerates, underground space is increasingly used, and the layout of facilities with different buried depths and texture of materials is intricate, which increases the difficulty of positioning and protection. Especially in old urban areas, the lack of accurate archives makes the risk of excavation damage higher [2]. According to the latest report from the Common Ground Alliance (CGA), approximately 189,549 underground facility damage incidents occurred in 2023, with sewer-related construction accounting for the largest proportion [3]. Therefore, developing effective methods for detection and location of underground pipelines is critical to preventing accidents at construction sites.

Ground-penetrating radar (GPR) may be the preferred approach for locating and mapping underground objects and pipelines [4]. It works by emitting, receiving, and processing high-frequency electromagnetic (EM) waves. However, it barely operates in adverse environments such as muddy or uneven ground and wet soil, which seriously weaken the EM wave penetration ability. Moreover, the active detection manner makes the system bulk and costly. So alternative methods such us passive magnetic detection technique is indispensable, which is immune to non-magnetic media and their boundaries. In fact, magnetic detection techniques have been extensively applied in various domains, including geological exploration [5], target identification [6], archaeological exploration [7], and airborne magnetic surveying for both military and civilian purposes [8,9,10].

Due to the presence of ferromagnetic materials in most underground pipelines, magnetic detection technology has been widely applied to detect various pipeline types [11,12,13], including those transporting crude oil, refined oil, natural gas, and water [14]. This technology does not require excitation and has a fast detection speed, making it suitable for the rapid detection of long-distance pipelines [15]. Magnetic flux leakage detection represents an important application of magnetic sensing technology for ferromagnetic pipelines, primarily serving the purpose of structural damage assessment and defect localization [16,17,18].

Some forward-modeling schemes have been proposed to analyze the magnetic anomalies generated by pipelines, such as the magnetic dipole reconstruction (MDR) method presented in [19,20,21]. To accelerate the computation and enhance the accuracy of the MRD method, a few segmented partitioning strategies were suggested [22,23]. Another modeling approach involves calculating the magnetic anomalies of ferromagnetic pipelines based on the Poisson equation, which governs the gravitational field and magnetic field. In [24], the characteristics of gravity and magnetic anomaly curves for parallel pipelines were analyzed, while in [25], deep learning neural networks were utilized to extract the magnetic anomalies of individual pipelines. In [26], a theoretical model for calculating the total-field magnetic anomalies of submarine pipelines was developed based on Poisson’s equation. The analysis showed a minor dependence on geomagnetic declination and a strong sensitivity to the pipeline azimuth.

Built on forward modeling, the adaptive mutation particle swarm optimization (AM-PSO) algorithm was applied in [27] to invert the parameters and burial depth of underground pipelines. In [28], an inversion scheme was established based on MDR using both piecewise segmentation and sectional segmentation methods for comparative analysis. Furthermore, a magnetic data-based method was developed for positioning buried pipelines by combining tilt angle analysis with downward continuation [29].

Considering the influence of the detection height on magnetic signals, relevant studies have been conducted. In [30], the magnetic field produced by a steel pipeline was measured at different internal pressures and detection heights, and the results show that the magnetic signals exhibited a second-order power attenuation with increasing detection height within 0.1–1.0 m. In [31], a spatial model for non-contact magnetic signals above ferromagnetic pipelines was established, and a correction method to eliminate the influence of detection height was proposed. In [32], a magnetic gradient method was developed to detect the buried depth of underground pipelines. In [33], magnetic field sensors were employed to remotely detect the horizontal position and vertical depth of underground power cables. The deployment of sensors for these conventional row or line scanning is usually sparse, resulting in limited spatial resolution and localization accuracy.

From the aforementioned works above, we notice that the forward modeling methods rely on a critical assumption: the magnetic moment distribution of ferromagnetic pipelines is uniform along the axial direction. However, this is not the general case due to factors such as pipeline manufacturing processes and complex local geomagnetic environments. Moreover, pipelines exhibit magnetic anisotropy such that the easier magnetization axis aligns along the axial direction [34], further complicating accurate modeling of pipeline magnetization. We also notice that their inversion methods are computationally complex and time-consuming, making it difficult to provide real-time location results at excavation sites. To meet real-time application, low-cost magnetometer sensors were suggested for installation on excavators, but only a principal demonstration in laboratory settings was presented, without verification of the entire system in real field scenarios [35,36].

Regarding the acquisition of magnetic field data, existing measurements usually adopt point-by-point or line-by-line scanning by translationally moving the sensors, which is inefficient and hard to operate on uneven or muddy ground. A more efficient circular scanning mode should be worth trying, which may also bring out novel localization methods.

In this study, we aim to develop a viable magnetic sensing system for detection of underground ferromagnetic pipelines and live cables. It is supposed to be low-cost but highly efficient in measuring operation and locating method. To meet the low-cost requirement, we used cheaper fluxgates instead of expensive atomic magnetometers. To facilitate the measuring operation, we adopted rotating scanning rather than row or line scanning. To avoid a complex location algorithm that requires reliable forward modeling and accurate measured data, we estimated the horizontal offset and strike angle of a possible pipeline by simply checking the periodic variation of the measured values due to the circular measurement course. Specifically, the main innovative points of this paper are the following:

(1) Integration of sensor nodes: Each node can in real time collect the magnetic field at the node place, as well as the position and attitude information of the node, and wirelessly transmit all collected data to a working laptop.
(2) Rotating scanning mode: Instead of the commonly used row or line scanning mode, we adopted rotating scanning, which is much more efficient to probe a specified area and applies to uneven or muddy ground.
(3) Simple localization method: We did not need complex forward modeling or accurate measured values but simply inspected the periodic variations of measured data to obtain the horizontal offset and strike angle, which was very fast and robust.

## 2. Methodology

This section describes the composition and operation of the measurement system, the theoretical analysis of pipeline magnetic anomalies, and the method to estimate the location and strike angle of a possible pipeline.

### 2.1. Composition of Measurement System

**Figure 1:**
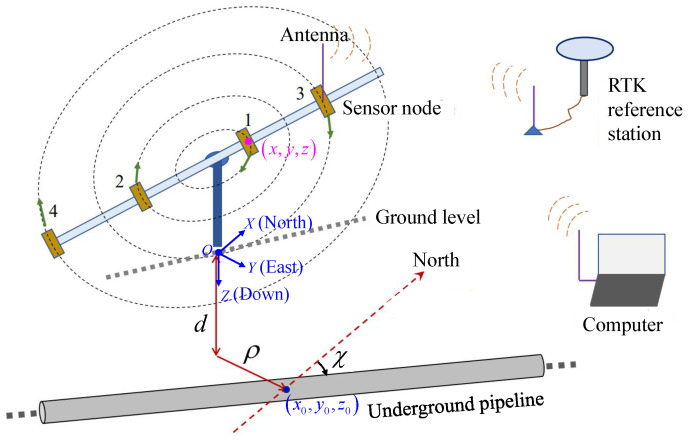
Illustration of the underground ferromagnetic pipe detection using a rotable magnetic sensor array.

The proposed measurement system is illustrated in Figure 1. It contains (1) at least one sensor node, while four nodes are shown in the figure; (2) a nonmagnetic rotable supporting structure that permits the sensor nodes to circularly measure the magnetic anomaly caused by a possible underground pipeline; and (3) a working laptop that wirelessly receives the data from each node and performs data analyses.

**Figure 2:**
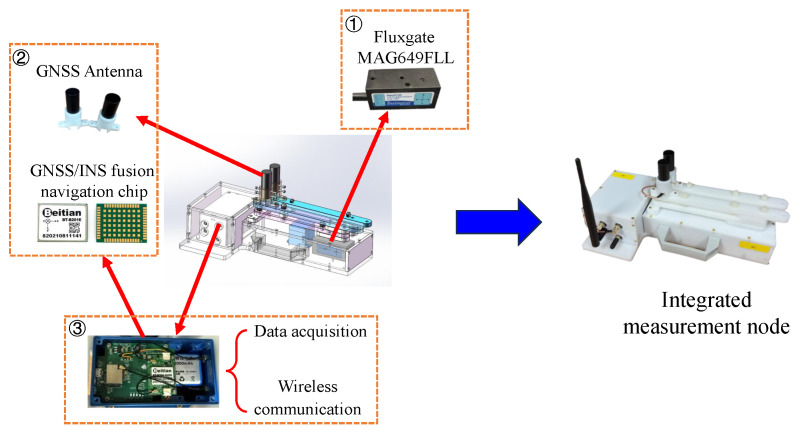
Specific components of the rotating magnetic sensors array survey system.

As shown in Figure 2, each node includes three main components: (i) a fluxgate, such as MAG649FLL (Bartington Instruments Ltd., Oxford, UK), to sense the magnetic field; (ii) a GNSS/INS fusion chip to record the position and attitudes of each node, which may use a RTK (real-time kinematic) reference station to increase the precision; and (iii) a combined data acquisition and wireless communication card to collect the data and transmit them to the laptop. In addition, a 3S lithium battery pack is integrated as the power supply. The specifications of components are summarized in Table 1.

### 2.2. Theoretical Analysis of Pipeline Magnetic Anomalies

First, we derive the magnetic anomaly distribution of the pipeline to provide a theoretical basis for the subsequent positioning algorithm. As illustrated in Figure 1, a right-handed coordinate system is established with the *x*-axis pointing north, the *y*-axis pointing east, and the *z*-axis downward. The pipeline is assumed to be horizontally laid and infinitely long. Given that the pipe diameter is much smaller than the distance between the pipeline and the measurement point, the pipeline can be considered as a straight line, i.e, its diameter is negligible. The pipeline is buried at a depth *H* below the surface, with a strike angle χ relative to geographic north and a horizontal offset ρ. Letting $r_{0}$$=(x_{0},y_{0},z_{0})$ be the center point of the pipeline, we have

(1) $$x_{0}=-\rho \sin\chi ,\;y_{0}=\rho \cos\chi ,\;z_{0}=-H.$$

Let $m_{0}$ denote the magnetic moment per unit length, not matter of the pipeline material. At the observation point r=(x,y,z), the magnetic-type Hertz potential generated by the pipeline is

(2) $$\Pi_{m}\left(r;r_{0}\right)=\frac{\mu_{0}}{4\pi }\int_{-\infty }^{\infty }\frac{m_{0}dl}{R}$$

where *R* is the distance from the elemental source point $r^{\prime }=r_{0}+l\left(\hat{x}\cos\chi +\hat{y}\sin\chi \right)$ to the observation point r, i.e.,

(3) $$\begin{array}{cc} R & =r-r_{0}-\left(\hat{x}\cos\chi +\hat{y}\sin\chi \right)l \end{array}$$

(4) $$\begin{array}{cc} R & =\left|R\right|=\sqrt{{\left(l-d\right)}^{2}+{\left|r-r_{0}\right|}^{2}-d^{2}} \end{array}$$

(5) $$\begin{array}{cc} d & =\left(x-x_{0}\right)\cos\chi +\left(y-y_{0}\right)\sin\chi \end{array}$$

in which *d* is the projection of $\left(r-r_{0}\right)$ along the pipeline direction $\left(\hat{x}\cos\chi +\hat{y}\sin\chi \right)$. The corresponding magnetic induction intensity can be found to be

(6) $$B_{pipe}\left(r;r_{0}\right)=\nabla \left(\nabla \cdot \Pi_{m}\right)=\frac{\mu_{0}}{4\pi }\bar{G}\left(r;r_{0}\right)\cdot m_{0}$$

with

(7) $$\begin{array}{cc} \bar{G}\left(r;r_{0}\right)=\frac{2}{D^{2}} & (\frac{2}{D^{2}}\left[\begin{array}{ccc} D_{x}D_{x} & D_{x}D_{y} & D_{x}D_{z}\\ D_{y}D_{x} & D_{y}D_{y} & D_{y}D_{z}\\ D_{z}D_{x} & D_{z}D_{y} & D_{z}D_{z} \end{array}\right]\\ \; & -\left[\begin{array}{ccc} 1-\cos^{2}\chi & -\cos\chi \sin\chi & 0\\ -\cos\chi \sin\chi & 1-\sin^{2}\chi & 0\\ 0 & 0 & 1 \end{array}\right]) \end{array}$$

and

(8) $$\left\{\begin{array}{c} D_{x}=x-x_{0}-d\cos\chi \\ D_{y}=y-y_{0}-d\sin\chi \\ D_{z}=z-z_{0} \end{array}\right.$$

(9) $$D^{2}=D_{x}^{2}+D_{y}^{2}+D_{z}^{2}={\left|r-r_{0}\right|}^{2}-d^{2}.$$

The measured magnetic field vector and its magnitude are

(10) $$B_{m}=B_{geo}+B_{pipe},\;|B_{m}|\approx |B_{geo}|+{\hat{B}}_{geo}\cdot B_{pipe}$$

where $B_{geo}$ is the local geomagnetic field, with ${\hat{B}}_{geo}$ denoting its direction, and $\left|B_{geo}|\gg |B_{pipe}\right|$ is assumed.

**Figure 3:**
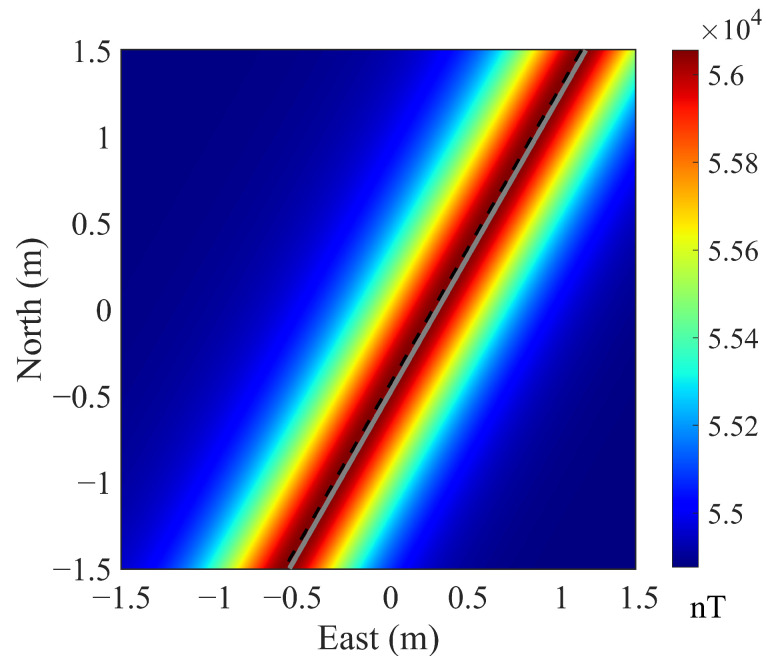
Simulated magnetic anomaly distribution of the pipeline.

Based on the formulae above, a series of simulation experiments were carried out to compute the magnetic anomaly distribution induced by the pipeline. The pipeline was placed centered at $r_{0}=\left(x_{0},y_{0},z_{0}\right)=\left(-0.15,0.26,-0.5\right)$ m, with a horizontal offset ρ=0.3 m and strike angle χ = 30°. We set $B_{geo}=\left[2.78,-0.39,4.72\right]\times 10^{4}$ nT (geomagnetic field in Beijing area), and assumed $m_{0}=10^{4}\times \left[B_{geo}+\left|B_{geo}\right|\left(\hat{x}\cos\chi +\hat{y}\sin\chi \right)\right]$, where the two terms represent the geomagnetic-induced and permanent magnetizations of the pipeline, respectively. The resulting magnetic distribution on the ground surface is shown in Figure 3, where the gray line denotes the true projection of the pipeline on the ground surface, and the black dashed line is drawn along the maximum value of magnetic anomaly. It can be seen that the maximum value is not situated directly above the pipeline but shifts a bit because of the last term in (10), which reflects the complex effects of the magnetic moment $m_{0}$, the horizontal offset ρ and strike angle χ, the radius vector R, and the direction of the geomagnetic field ${\hat{B}}_{geo}$.

### 2.3. Rotating Scanning Measurement

**Figure 4:**
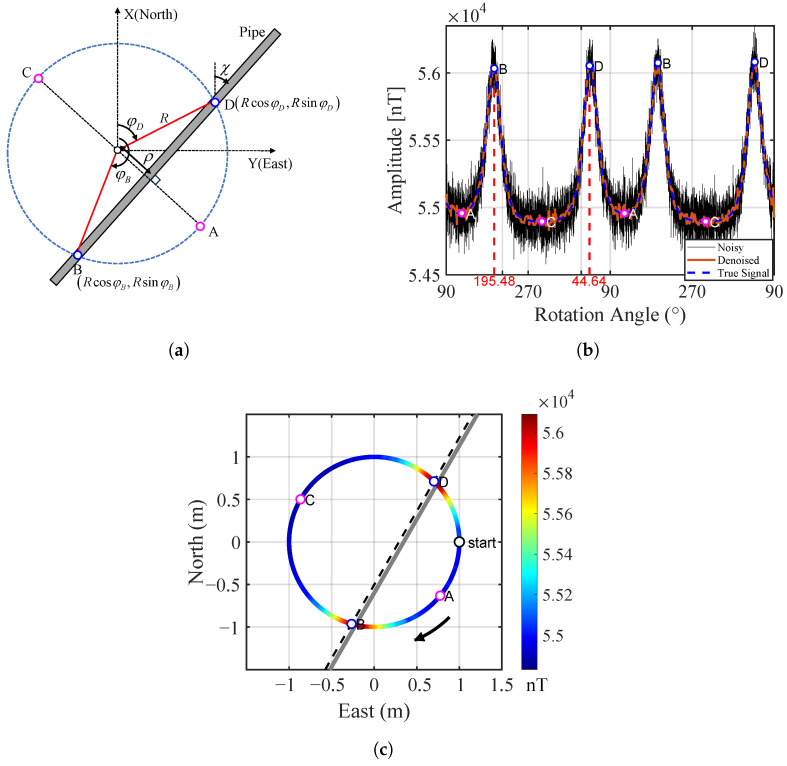
(**a**) Illustration of the detection operation and estimates of strike angle and offset of a pipe. (**b**) Comparison of noisy, clean, and denoised total magnetic field signals. (**c**) Localization result of the pipeline using denoised signal maxima.

Referring to Figure 4a, when a node is measuring the magnetic field cyclically along a circular course, the measured values varies cyclically, too. It is apparent that the extreme values in one turn will appear roughly at the four A, B, C, and D positions. Because the measured values include both the local geomagnetic field and the magnetic anomaly caused by the pipe, the extreme values should not be exactly but roughly at the four positions. By connecting the points B and D, the strike angle of the pipe, as well as its offset from the center of the rotating rail, can be estimated. We can estimate them just using the cyclically varying property without accurate magnetic field values, so we can use a low-cost fluxgate rather than an expensive atomic magnetometer. To increase the reliability, multiple sensor nodes may be employed to form an array, as illustrated in Figure 1.

In addition, based on the rotational measurement scheme and the simulation parameters given in Section 2.2, a single sensor is placed on the ground and rotates two full turns around the origin with a radius of 1 m. To simulate the environmental magnetic noise, zero-mean Gaussian white noise with a standard deviation of 100 nT was added to the clean signal. Subsequently, an adaptive soft-thresholding denoising method based on wavelet decomposition was applied. The signal was decomposed into five levels using the “db4” wavelet to extract its multi-scale features, which effectively suppressed noise while preserving the main signal components. The denoising performances were quantitatively evaluated using the signal-to-noise ratio (SNR) and the correlation coefficient (CC), defined as

(11) $$\mathrm{SNR}=10\log_{10}\frac{\sum_{i=1}^{N}s_{i}^{2}}{\sum_{i=1}^{N}{\left(s_{i}-x_{i}\right)}^{2}}$$

(12) $$\mathrm{CC}=\frac{\sum_{i=1}^{N}\left(s_{i}-\bar{s}\right)\left(x_{i}-\bar{x}\right)}{\sqrt{\sum_{i=1}^{N}{\left(s_{i}-\bar{s}\right)}^{2}\sum_{i=1}^{N}{\left(x_{i}-\bar{x}\right)}^{2}}}$$

where *N* is the total number of samples; $s_{i}$ is the clean signal sample; $x_{i}$ denotes the noisy or denoised signal sample before or after processing, respectively; and $\bar{s}$ and $\bar{x}$ are their corresponding mean values.

The simulation results are shown in Figure 4b, where the black solid line represents the noisy total magnetic field, the blue dashed line represents the clean signal, and the orange solid line indicates the denoised signal. After denoising, the SNR increases from 11.0 dB to 24.9 dB, and the CC improves from 0.963 to 0.998, demonstrating a significant enhancement in signal quality. The local maxima of the total magnetic field were extracted from the denoised signal (blue points) and connected, then displayed on the scanning plane z=0, as shown in Figure 4c. The gray solid line represents the true pipeline placement. It can be observed that the maxima positions are situated almost directly above the pipeline, with a strike angle error of 0.54° and an offset error of 0.034 m.

**Figure 5:**
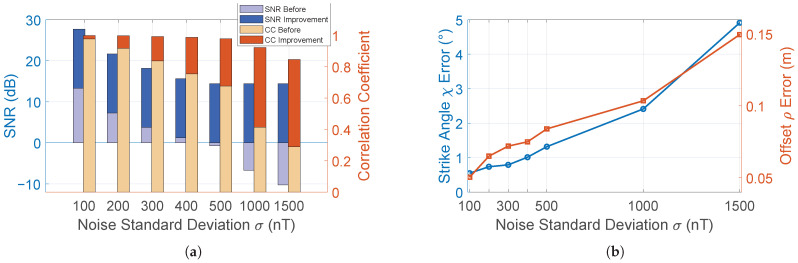
Effect of noise on magnetic signal denoising and pipeline localization. (**a**) Signal quality (SNR and CC) before and after denoising. (**b**) Localization error (strike angle and offset).

To further evaluate the robustness of the wavelet-based denoising method under different noise levels, Gaussian white noise with varying standard deviations was added to the clean signal. As shown in Figure 5a with 1000 realizations, as the noise intensity increases, the improvement in SNR remains stable at approximately 14 dB, while the improvement in CC also increases significantly; of course, the location errors of the strike angle and horizontal offset increase mildly as well, as shown in Figure 5b.

We may emphasize that adopting rotating rather than translational scanning is advantageous for two reasons. First, the efficiency is much higher when scanning the same area, not to mention the difficulty in moving the nodes on rough or muddy ground. Second, we can judge the existence and strike angle of a pipeline based on the periodic variations without using accurate magnetic anomaly data that are hard to separate from a complex background geomagnetic field and measurement errors.

**Figure 6:**
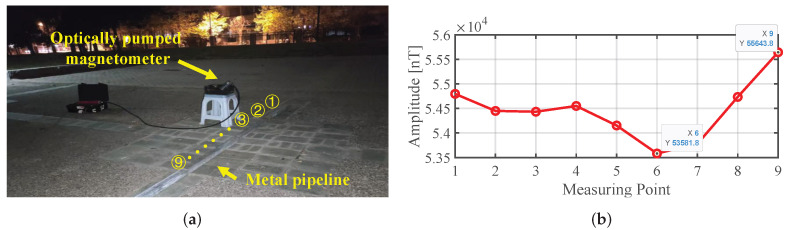
Experimental verification of non-uniform magnetization of a general ferromagnetic pipe. (**a**) Field measurement scene. (**b**) Measured magnetic anomalies at nine positions along the pipe.

Another point worth noting is that in the analysis above, the measured values at points B and D in Figure 4 should be the same if the magnetization along the pipeline is uniform. However, this may not be the general case, that is, the pipeline is not uniformly magnetized during the manufacturing process. So the measured values at the points B and D are usually not equal. To verify this fact, an experiment was conducted, as illustrated in Figure 6a. A long ferromagnetic pipeline was pulled to move near an optical-pumping magnetometer (Cs-3 Cesium Magnetometer, Scintrex Ltd., Concord, ON, Canada) with a sensitivity of approximately $0.0006\;\mathrm{nT}/\sqrt{\mathrm{Hz}}$ (rms) and a dynamic range of 15,000–105,000nT (please note, we used a high-precision atomic magnetometer instead of a low-precision fluxgate solely for this verification). The measured values when the nine points on the pipeline were closest to the magnetometer are plotted in Figure 6b. The slant distance from the magnetometer to the pipe was 46 cm, while the spacing of the nine points was 25 cm. The results show that the maximum value at point 9 is greater than the minimum value at the point 6 by 2062 nT, which reflects a pronounced non-uniformity in the magnetization along the pipe. This finding indicates that any inversion algorithm based on forward modeling with a uniformly magnetized assumption may not align with real ferromagnetic pipelines.

### 2.4. Estimate of Pipeline Location and Strike Angle

#### 2.4.1. Horizontal Placement Estimation

When a ferromagnetic pipeline is present underground, the magnetic field time series collected by the rotating magnetic sensor nodes typically exhibit periodic characteristics. The prominent extreme points in the data correspond to the positions where the sensor attains the closest proximity to the pipeline during its rotational course. These periodic variations are directly related to the number of rotations. By recording the rotated angles corresponding to the extreme points, the approximate orientation of the underground pipeline can be quickly inferred.

By extracting the latitude and longitude information of the detection nodes at the occurrence of these extreme points, which is obtained by the real-time kinematic (RTK) module installed on the nodes, hidden pipeline locations beneath the nodes can be identified. Subsequently, fitting all candidate points where pipelines are suspected underneath into a straight line allows for determinations of the pipeline’s orientation and horizontal offset coordinates.

Let the coordinates of all candidate points be denoted as $V=\{\left(x_{1},y_{1}\right),\left(x_{2},y_{2}\right),\ldots ,\left(x_{C},y_{C}\right)\}$, where each $\left(x_{i},y_{i}\right)$ corresponds to a position where the magnetic field reaches an extreme value. The linear fitting procedure is based on the least squares method, aiming to find the best-fit line y=ax+b, where *a* is the slope and *b* is the intercept. The parameters *a* and *b* are determined by minimizing the sum of the squared errors:

(13) $$\min_{a,b}\sum_{i=1}^{C}{\left[y_{i}-\left(ax_{j}+b\right)\right]}^{2}.$$

This optimization problem can be solved using standard linear regression techniques to obtain the optimal values of *a* and *b*. The resulting fitted line determines the pipeline strike angle χ and the pipeline offset coordinates:

(14) $$\chi =\tan^{-1}\left(a\right)$$

(15) $$\left(x_{0},y_{0}\right)=\left(-\frac{ab}{1+a^{2}},\;\frac{b}{1+a^{2}}\right).$$

The quality of the linear fit can be evaluated using the coefficient of determination $R^{2}$, defined as

(16) $$R^{2}=1-\frac{\sum_{i=1}^{C}{\left(y_{i}-{\hat{y}}_{i}\right)}^{2}}{\sum_{i=1}^{C}{\left(y_{i}-\bar{y}\right)}^{2}}$$

where ${\hat{y}}_{i}$ denotes the predicted value from the fitted line, and $\bar{y}$ represents the mean of the observed values. The value of $R^{2}$ ranges between 0 and 1, with a value closer to 1 indicating a better fitting.

The magnetic sensor array continuously rotates around a central point to capture magnetic anomalies from underground pipelines. We may display the measured values at the positions where they are collected by the nodes to form a diagram, using a color bar to indicate the intensities. The extreme values of magnetic fields will fall in a belt that should roughly show the pipeline’s orientation.

#### 2.4.2. Buried Depth Estimation

For a horizontally buried pipeline within the measurement area, the burial depth d(d>0) can be estimated by measuring magnetic field variations at *M* distinct heights $h_{i}\;\left(i=1,2,\ldots ,M,h_{i}>0\right)$ above the ground. The total magnetic field response of the pipeline at height $h_{i}$ is defined as

(17) $$\Delta T_{i}=\left|T_{i}^{\mathrm{extreme}}-T_{\mathrm{geo}}\right|,\;i=1,2,\ldots ,M$$

where $T_{i}^{\mathrm{extreme}}$ represents the extreme value of the total field at height $h_{i}$ obtained through averaging multiple rotation cycle extrema to improve the measurement stability, and $T_{\mathrm{geo}}$ denotes the background geomagnetic field measured as the mean value in pipeline-free regions.

The depth estimation follows from the magnetic field decay relationship

(18) $$\Delta T_{i}=k{\left(h_{i}+d\right)}^{-\alpha },\;i=1,2,\ldots ,M$$

where *k* is a proportionality constant that reflects the material properties and dimensional characteristic of the pipeline, $h_{i}+d$ represents the vertical distance between the sensor measurement point and the pipeline, and α reflects the rate at which the magnetic field gradient changes with the vertical distance between the measurement point and the pipeline. Each node independently measures the magnetic field gradient at its corresponding location without interference between the nodes.

By applying a nonlinear fitting method, the values of *k* and *d* can be determined by minimizing the following objective function:

(19) $$\min_{k,d}\sum_{i=1}^{M}{\left[\Delta T_{i}-k{\left(h_{i}+d\right)}^{-\alpha }\right]}^{2}.$$

**Figure 7:**
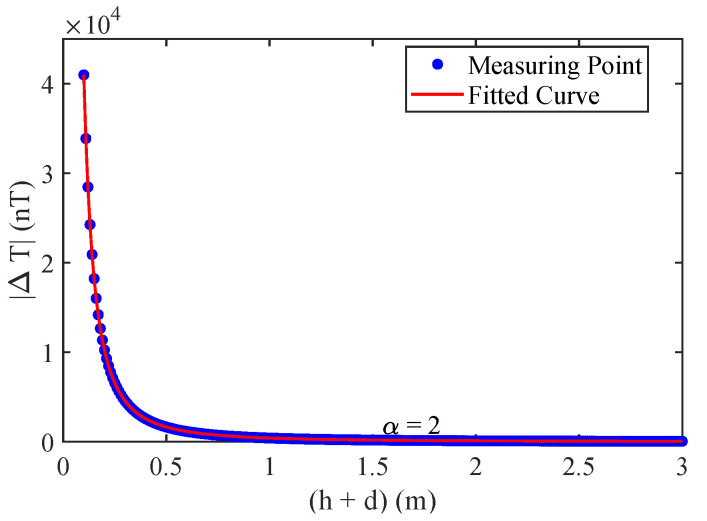
Simulated magnetic field attenuation versus vertical distance, with α=2.

This optimization problem can be solved using intelligent optimization algorithms, and here the Particle Swarm Optimization (PSO) [37] was used. Based on the simulation parameters presented in Section 2.2, the magnetic field at the point $\left(x_{0},y_{0},0\right)$ was computed for various burial depths ranging from 0.1 m to 3 m. The resulting values were then fitted using the model of (18), as shown in Figure 7, yielding an optimal factor α=2 to indicate second-order power attenuation behavior.

## 3. Field Experimental Results

This section evaluates the designed rotating magnetic sensor array through field experiments. First, we took an iron pipe as the detection target, with various target and sensor positions. Multiple measurements were conducted, and the proposed methods were applied to give the localization results. Last, the integrated system and algorithm were validated through detecting a real ferroconcrete pipeline.

### 3.1. Experimental Setup

The experiments were conducted at an outer suburban place of Beijing (N 40°01’, E 117°08’), where the local geomagnetic field strength was 54,586 nT, with a gentle fluctuation of 1 nT during one minute caused by the undulating ionosphere. The target was a square iron pipe that measured 6 m in length and 0.05 m in outer diameter, which was employed to simulate an underground ferromagnetic pipeline. To facilitate the measurement and repositioning, the pipe was placed directly on the flat ground rather than buried underground.

**Figure 8:**
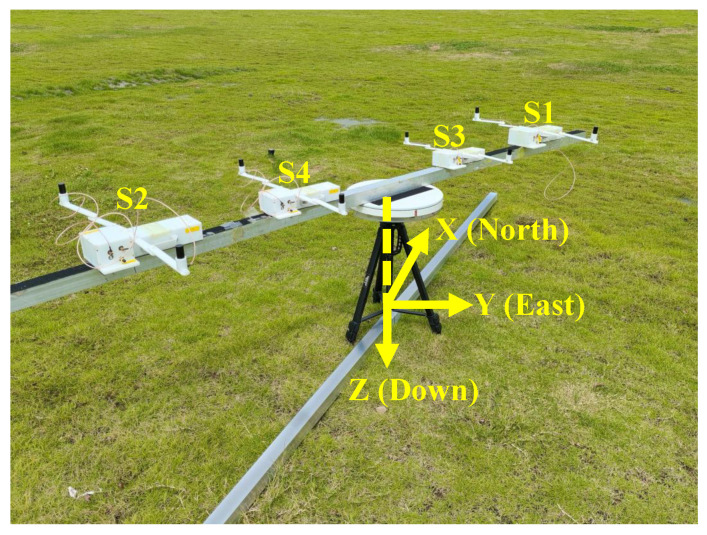
Experimental setup using a square steel tube with an outer diameter of 5 cm to simulate an underground pipeline.

As illustrated in Figure 8, the coordinate system was established with the projection of the array center on the ground as the origin *O*. The *x*-axis aligned with geographic north, the *y*-axis pointed east, and the *z*-axis was downward, while the ground plane corresponded to z=0. Initially, the background magnetic field environment was recorded in the absence of the target as a reference baseline. Subsequently, the array was fixed at a height of h=0.55 m above the ground to measure the magnetic field distribution when the target pipe was positioned at three different locations. Specifically, the pipe was oriented at a fixed strike angle *χ* = 15° (relative to geographic north), with its central axis initially passing through the origin of the coordinate system. The pipe was then shifted 0.5 m to the east and west, respectively. Finally, the height of the array above the ground was adjusted to study the variation of magnetic field distribution with height, which was used to estimate the pipe’s burial depth. Through these experimental procedures, a total of nine measurements were performed (the pipe was placed at three positions, and for each place, the measurements were carried out at three heights).

### 3.2. Pipeline Location Results

**Figure 9:**
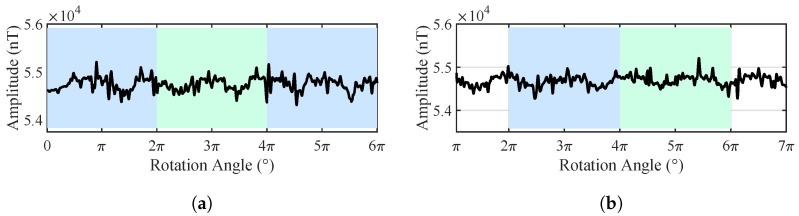
Time-domain diagram of the total magnetic field output from two nodes in the absence of pipeline target. (**a**) Node 1. (**b**) Node 2.

When no pipeline was present, the outputs of the measurement nodes predominantly reflected the variations in the background magnetic gradients, as shown in Figure 9. Due to the manual operation, the non-uniform rotational speed introduced significant spikes, but no periodicity was observed in the three turns, indicating no pipe exists.

**Figure 10:**
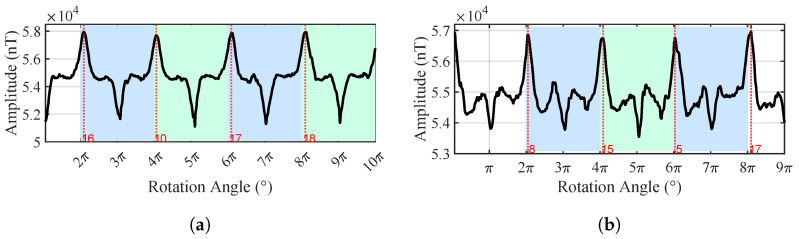
Time-domain diagram of the total magnetic field output from two nodes with the pipeline target at the center (*h* = 0.55 m). (**a**) Node 1. (**b**) Node 2.

In the presence of a pipeline target, the magnetic field data acquired by the nodes exhibit clear periodic variations, as shown in Figure 10. Several prominent extrema appear in the magnetic field curves when the nodes pass the position almost directly above the pipeline. For instance, when the rotation angles of Node 1 are 16°, 10°, 17°, and 18°, the magnetic field intensity reaches local maxima, and the corresponding node positions are in close proximity to the pipeline. The dispersion in these angles is mainly caused by GPS positioning errors, fluxgate measurement errors, and asynchronous sampling; in practice, we usually perform several cycles and take the average to reduce the error.

**Figure 11:**
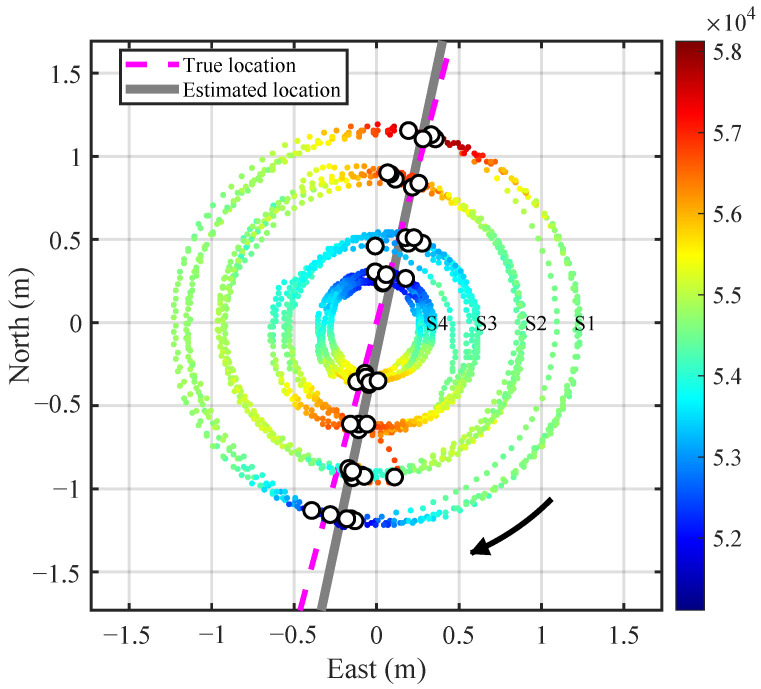
Localization results with the pipeline target at the center (*h* = 0.55 m). The colored points indicate the coordinates where the four nodes collect data along circular courses, where the color reflects the magnetic field intensity at the positions. The small white circles mark the coordinates where the magnetic anomaly reach extreme values. The gray solid line and magenta dashed line show the estimated and true pipeline placements, respectively.

In the localization experiment, the coordinates of all significant extrema points from the four sensor nodes were recorded. A linear fitting was then applied in the XOY plane to estimate the pipeline orientation and offset coordinates. All the extrema points for each node at three heights and rotating for four turns are included in the fitting. The results are shown in Figure 11. The points are colored according to the magnetic field intensities at the measurement positions. The small white circles denote the magnetic field extrema recorded by the four nodes. The estimated pipeline is shown as a gray solid line, while the actual pipeline is indicated by a magenta dashed line. Though some orientation discrepancy exists, it meets common engineering application needs.

**Figure 12:**
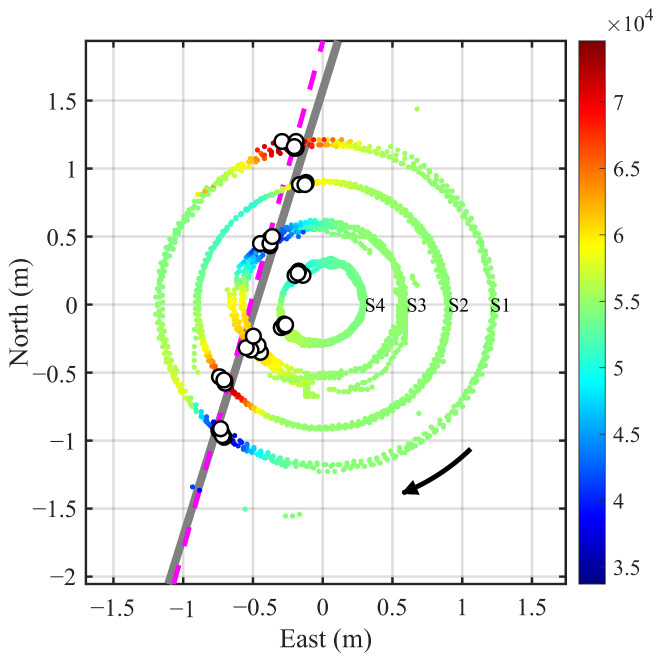
Localization results with the pipeline on the west side (*h* = 0.25 m).

**Figure 13:**
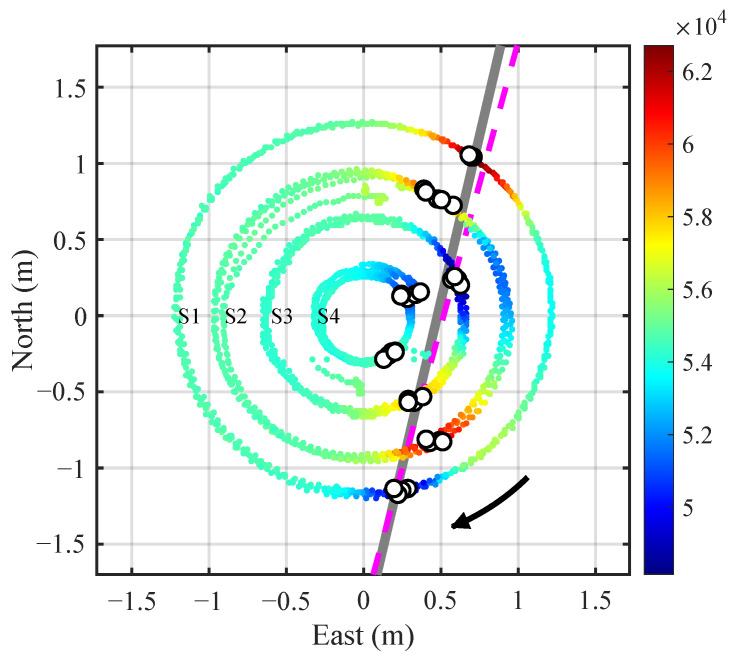
Localization results with the pipeline on the east side (*h* = 0.40 m).

Similarly, the localization results for the pipe shifted to the west and east a distance are displayed in Figure 12 and Figure 13, respectively. The results demonstrate a high consistency between the estimated and actual positions (orientation and offset) of the pipeline. It should be pointed out that in Figure 12 and Figure 13, the points for magnetic anomaly extrema occurring at Node 4 should be excluded from the linear fitting process because the node does not pass directly above the estimated pipeline even if the points are included in the fitting.

To measure the reliability of the linear fitting, the regression model coefficients *a* and *b* and the $R^{2}$ values are summarized in Table 2. The results show an average $R^{2}$ of 0.913, indicating a strong fitting performance and high stability and accuracy of the localization results.

**Figure 14:**
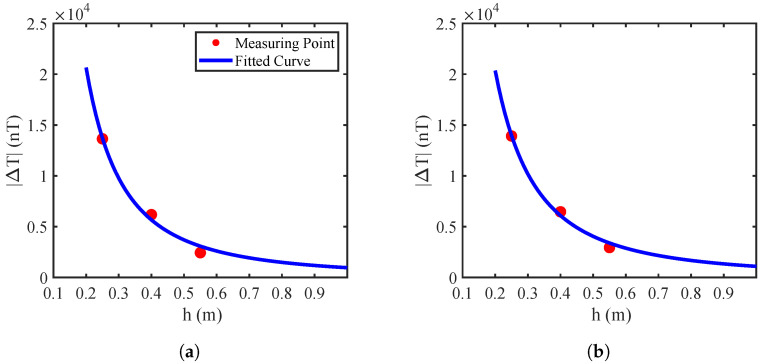
Curve-fitting results of burial depth estimation with the pipeline at the center. (**a**) Node 1. (**b**) Node 2.

To estimate the buried depth, we took the data in Figure 11 as an example, i.e., the obtained extrema points at three heights were substituted into (3) and (18) to solve for *k* and *d*. The nonlinear fitting results are given in Figure 14, where the red dots denote measured points, and the blue curve represents the fitted relation between the height *h* and the magnetic field gradient ΔT. Table 3 lists the coefficients of the nonlinear regression models and the $R^{2}$ values for each node, with an average $R^{2}$ of 0.9933, indicating an excellent fitting performance. The burial depth was estimated by each node independently, while the final pipeline depth was obtained by averaging the estimates from four nodes as the pipeline is was assumed to be horizontally placed.

Table 4 summarizes the pipeline localization inversion parameters under different experimental conditions. The measurements were carried out for three pipeline locations and for each location at three different heights above the ground. The inversion parameters include the pipeline strike angle χ (defined as the angle between the pipeline direction and geographic north), horizontal offset coordinates $\left(x_{0},y_{0}\right)$, and burial depth *d*. The results indicate a strike angle error of 2.68°, horizontal position errors of 0.016 m in the *x*-direction and 0.031 m in the *y*-direction, a burial depth error of 0.017 m, and a spatial slant-range error of 0.039 m. These results confirm that the proposed pipeline localization algorithm achieves satisfactory accuracy and stability.

### 3.3. Real-World Pipeline Localization Case

**Figure 15:**
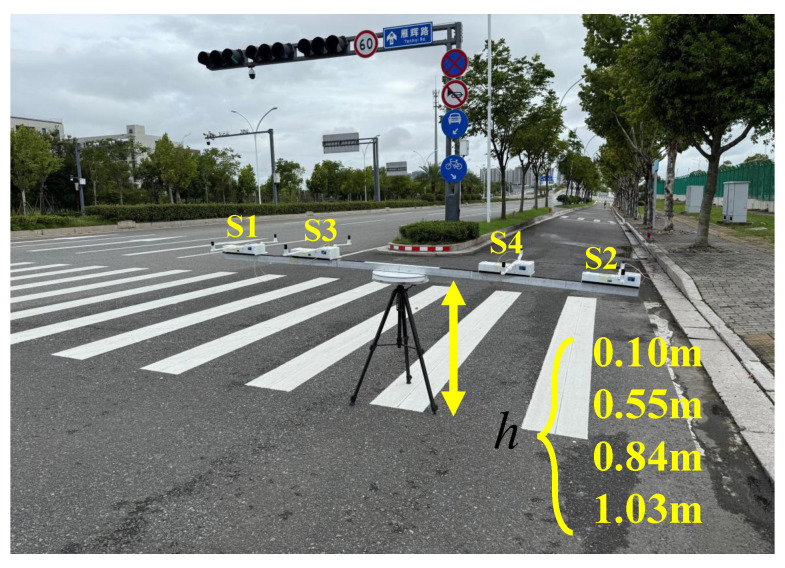
Real pipeline detection site.

To verify the applicability of the proposed system and localization algorithm in real pipeline environments, an underground reinforced concrete pipe with a diameter of 1.2 m and a burial depth of 3.0 m was selected as the detection target. The experiment was conducted on a service road along Yanhui Road, Dongtou District, Wenzhou (N 27°56’, E 120°56’). Four measurement configurations at different heights were selected, as illustrated in Figure 15.

**Figure 16:**
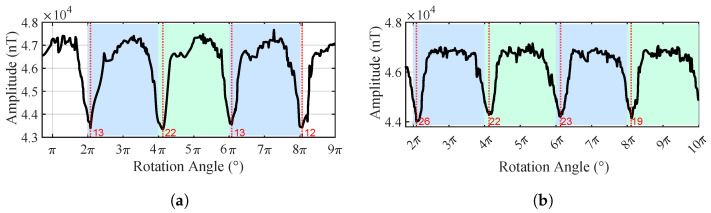
Time-domain diagram of the total magnetic field output from two nodes with the real pipeline (*h* = 0.1 m). (**a**) Node 1. (**b**) Node 2.

**Figure 17:**
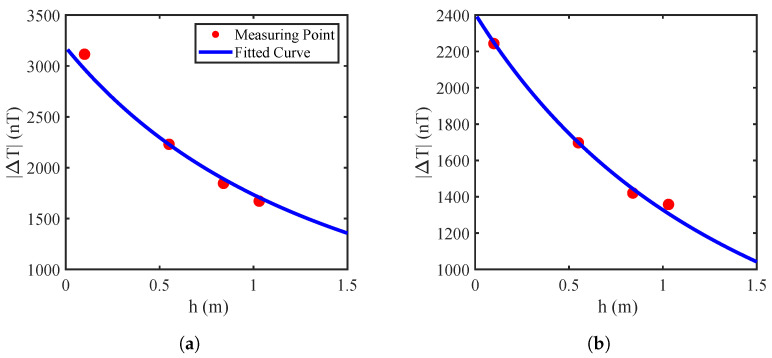
Curve-fitting results of burial depth estimation with the real pipeline. (**a**) Node 1. (**b**) Node 2.

**Figure 18:**
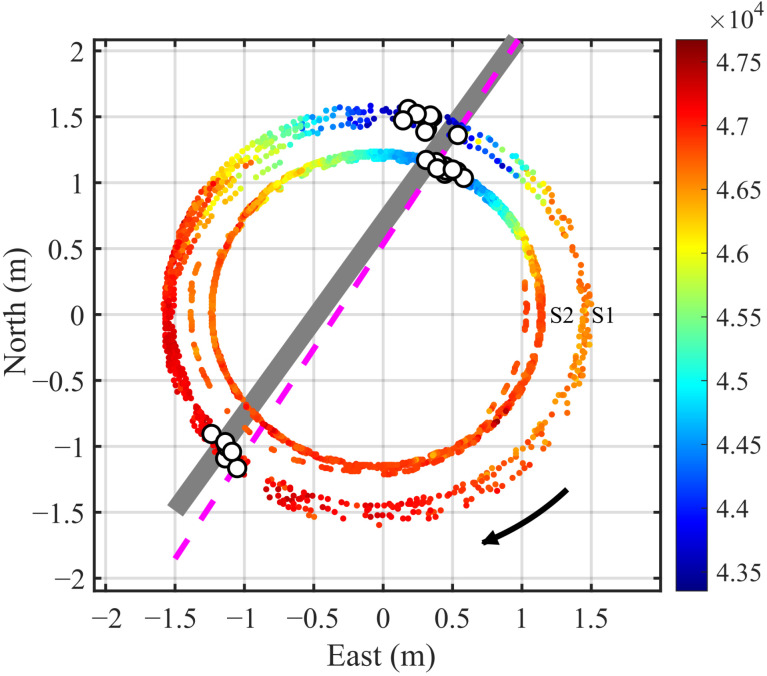
Localization result with the real pipeline (*h* = 0.1 m).

The experimental data show that the total magnetic field values measured by Nodes 1 and 2 rotating for four turns at the height of 0.1 m above the ground exhibit a pronounced periodic variation, as shown in Figure 16, indicating the presence of a linearly distributed magnetic anomaly target. The nonlinear fitting results for the burial depth estimation are shown in Figure 17. The fitted coefficients and $R^{2}$ values of the nonlinear regression model are listed in Table 5, with an average $R^{2}$ value of 0.9869, demonstrating an excellent fitting performance. Due to the large pipeline diameter and deep burial depth, magnetic field data from Nodes 1 and 2 with larger rotation radii were used for the linear fitting. As a rule of thumb, we require the rotation radius of a node to be greater than the half distance to the pipeline. Since the maxima from Node 2 are not distinguishable, the fitting ultimately incorporates both the maxima and minima from Node 1, and only the minima from Node 2. The horizontal localization result at h=0.10 m, as shown in Figure 18, indicates that the overall distribution remains approximately consistent with the pipeline’s orientation.

Table 6 summarizes the inversion parameters and errors at the four measurement heights, where the pipeline strike angle error is 5.21°, the horizontal localization errors are 0.144 m in the *x*-direction and 0.059 m in the *y*-direction, and the burial depth error is 0.15 m. The overall spatial slant-range localization error is 0.216 m, or a relative error of 7.2% compared with the buried depth of 3 m. These experimental results confirm that the proposed pipeline localization algorithm exhibits robust applicability and acceptable accuracy in real-world pipeline environments.

## 4. Concluding Remarks

This paper presents a portable wireless magnetic detection system for localization of underground ferromagnetic pipelines. The tripod and rail are retractable and lightweight at only a few kilograms. An integrated sensor node takes up about $30\times 10\times 10\;{\mathrm{cm}}^{3}$ and weighs 1.5 kg. It performs rotating scanning measurements driven by a micro motor, has a high measuring efficiency, and tolerates complex ground conditions. Moreover, the localization methods are simple and robust, as we only need to gather the coordinates on circular scanning courses where the measured magnetic field reaches extreme values. By fitting these coordinates with a straight line, we obtain the estimated pipeline placement. Both simulated and field experiments confirm the viability to give applicable localization results in real time. The realized localization errors of slant range and strike angle are a few decimeters and several degrees, respectively. Future works may follow regarding multiple aspects: first, improve the localization accuracy by incorporating some prior knowledge of pipelines and local environments; second, extend the application to multiple pipelines, armored cables, and discrete targets; third, accelerate the detection process by utilizing drones to carry the nodes; fourth, acquire high-precision data by using atomic magnetometers at increased cost to meet special demands.

## Figures and Tables

**Table 1:** System hardware specifications.

Specification	Value
**Magnetometer Module**	
Sensors	Bartington Mag649FLL
Measuring Range	±100 T
Orthogonality Error	<1° error between axes
Magnetic Field Resolution	0.01 nT
**GNSS/Inertial Measurement Unit (IMU) Modules**	
Sensors	BT-B201E
Accelerometer	±16 g
Gyroscope	±1000 deg/s
Satellite System	Beidou, GPS, Gallileo, Glonass
Horizontal Accuracy	≤8 mm ± 1 ppm
Roll/Pitch Accuracy	≤0.02° (1σ)
Yaw Accuracy	≤0.2° (1σ)
**Data Acquisition and Communication Card**	
Sampling Rate	10 Hz
Wireless Module	ESP32-WROOM-32 Wi-Fi

**Table 2:** Linear regression model coefficients using measurement values at three heights for three placements of the pipeline.

Height of Nodes Above Ground (m)	*a*			*b* (m)			$R^{2}$		
0.55	4.655	3.141	4.676	−0.169	1.539	−2.541	0.9479	0.8558	0.8694
0.40	4.464	2.990	4.663	−0.178	1.554	−2.238	0.8850	0.9288	0.9142
0.25	4.193	3.283	4.866	−0.176	1.577	−2.517	0.9403	0.9132	0.9620
Overall	4.501	3.138	4.735	−0.174	1.557	−2.432	0.913		

**Table 3:** Nonlinear regression model coefficients for different nodes with the pipeline at the center.

Node Index	*k* ($\mathrm{nT}\cdot m^{\alpha }$)	*d* (m)	$R^{2}$
1	1000.07	0.020	0.9895
2	1185.99	0.041	0.9945
3	1027.84	0.000	0.9947
4	1112.65	0.010	0.9945
Overall	1081.64	0.0177	0.9933

**Table 4:** Simulation pipeline experimental inversion parameters and errors.

Experiment Index		Inversion Parameters									
		*χ* (°)			*x*_0_ (m)			*y*_0_ (m)			*d* (m)
1	True	15			0			0			0
	Estimate	12.12	12.62	13.41	−0.007	−0.009	−0.009	0.035	0.038	0.040	0.018
	Error	2.88	2.37	1.59	0.007	0.009	0.009	0.034	0.038	0.040	0.018
2	True	15			0.129			−0.483			0
	Estimate	17.66	18.49	16.94	0.142	0.156	0.134	−0.445	−0.467	−0.440	0.014
	Error	2.66	3.50	1.94	0.013	0.027	0.005	0.038	0.015	0.043	0.014
3	True	15			−0.129			0.483			0
	Estimate	12.07	12.10	11.61	−0.111	−0.098	−0.102	0.520	0.459	0.496	0.018
	Error	2.93	2.90	3.39	0.018	0.031	0.027	0.037	0.024	0.013	0.018
Average error		2.68 ± 0.63			0.016 ± 0.010			0.031 ± 0.011			0.017 ± 0.002
					0.039 ± 0.015						

Note: Each column of χ, $x_{0}$, and $y_{0}$ corresponds to one of the three pipeline placements.

**Table 5:** Nonlinear regression model coefficients for different nodes with the real pipeline.

Node Index	*k* ($\mathrm{nT}\cdot m^{\alpha }$)	*d* (m)	$R^{2}$
1	25,318.39	2.82	0.9802
2	20,025.93	2.88	0.9936
Overall	22,672.16	2.85	0.9869

**Table 6:** Inversion parameters and errors of real pipeline experiments.

Experiment Index		Inversion Parameters												
		*χ* (°)				*x*_0_ (m)				*y*_0_ (m)				*d* (m)
1	True	32.14				0.204				−0.208				3.00
	Estimate	34.55	37.63	38.39	38.82	0.322	0.347	0.360	0.364	−0.222	−0.268	−0.286	−0.293	2.85
	Error	2.41	5.49	6.25	6.68	0.118	0.143	0.156	0.160	0.014	0.060	0.078	0.085	0.15
Average error		5.21 ± 1.93				0.144 ± 0.019				0.059 ± 0.032				0.15
						0.216								

## Data Availability

The original contributions presented in this study are included in the article. Further inquiries can be directed to the corresponding author.
